# Photoprotective potential in some medicinal plants used to treat skin diseases in Sri Lanka

**DOI:** 10.1186/s12906-016-1455-8

**Published:** 2016-11-24

**Authors:** Mayuri Tharanga Napagoda, Benthota Malavi Arachchige Shamila Malkanthi, Subasinghe Appuhamillage Kaumudi Abayawardana, Mohomed Mallique Qader, Lalith Jayasinghe

**Affiliations:** 1 Department of Biochemistry, Faculty of Medicine, University of Ruhuna, Galle, 80000 Sri Lanka; 2 National Institute of Fundamental Studies, Hantana Rd, Kandy, 20000 Sri Lanka

**Keywords:** Photoprotective, Sunscreen, Antioxidant, Medicinal plants

## Abstract

**Background:**

The constant exposure to solar ultraviolet radiation (UV) has a variety of harmful effects on human health. Although synthetic sunscreen products have been introduced as a preventive/therapeutic strategy, with the realization of their adverse side effects, the recent trend is to search for human friendly alternative formulations especially of plant origin. Therefore, the present study focuses on evaluation of photoprotective activity of aqueous extracts (1 mg/ml) of eleven medicinal plants in Sri Lanka that have been widely employed in traditional medicine as treatment options for various skin diseases and to improve the complexion.

**Methods:**

For the determination of UV filtering potential of the extracts, UV absorption was measured and the sun protection factor (SPF) was calculated according the Mansur equation. The antioxidant activity was evaluated by DPPH and ABTS assays.

**Results:**

Among the extracts, *Atalantia ceylanica*, *Hibiscus furcatus*, *Leucas zeylanica*, *Mollugo cerviana*, *Olax zeylanica* and *Ophiorrhiza mungos* have displayed SPF value ≥ 25, which are even higher than two commercial photoprotective creams used as reference compounds. *L. zeylanica* and *O. mungos* have displayed a high UV absorbance in 260–350 nm range indicating their potential of being broad spectrum sunscreens. In addition, the extract of *O. mungos* was found to be photostable, without any significant reduction in the SPF after exposure to direct solar radiation for 21 days. DPPH assay and the ABTS assay revealed that the extracts possess high antioxidant activity.

**Conclusion:**

The results of the present study suggest that the presence of secondary metabolites with antioxidant property could be responsible for the high UV absorbance. Our findings would offer an exciting avenue for further research towards the development of herbal cosmetics.

## Background

During the past few decades, the anthropogenic activities had led to a substantial damage to the protective ozone layer and resulted in a significant increase of the solar radiation reaching the earth. As a consequence, the incidence of various diseases and disorders related with the excessive exposure to solar ultraviolet (UV) radiation has alarmingly increased over the recent years [1]. Solar UV radiation is classified into three major categories depending on the wavelength; UV-A (320–400 nm) UV-B (290–320 nm) and UV-C (200–290 nm). Intense or overexposure to UV-A and UV-B leads to sun burn, erythema, inflammation, hyperpigmentation, wrinkling, hyperplasia, local immunosuppression, photoaging and photocarcinogenesis. Although UV-C is most biologically damaging, it gets effectively filtered by the ozone layer [2–4].

The UV exposure to the skin leads to the generation of free radicals/reactive oxygen species which exert deleterious effects by oxidizing biologically essential molecules and induced oxidative damage. The excess of free radicals results in a cascade of events for example, mitogen-activated protein kinase cascade, induction of heme oxygenase-1 and matrix metalloproteinase in the skin and thereby mediating progressive deterioration of cellular structure and function as well as modification of DNA and abnormal expression of cellular genes. Although the skin possesses an elaborate antioxidant system to deal with the UV induced oxidative stress, the extensive and chronic exposure to UV could exceed the cutaneous antioxidant capacity, hence leads to oxidative damage causing variety of harmful effects in the skin [5].

As it would be impractical to reduce the excessive exposure to solar radiation, a novel approach called “photochemoprevention”/“photoprotection” has been introduced to overcome the detrimental effects caused by UV radiation. It involves the use of various photochemopreventive/photoprotective agents which functions via prevention of the damage caused by UV radiation and/or modulation of different cellular responses to UV radiation to prevent, stop or correct tumour promotion and progression. The most popular strategy in the present day practice to reduce the amount of UV radiation penetrating the skin is the topical application of sunscreen products that contain UV absorbing, reflecting or scattering active molecules. Sunscreens with a sun protective factor (SPF) value of 15 or greater are highly recommended and these compounds are incorporated in several cosmetic products such as creams, gels, oils and lotions [6]. Octylmethoxycinnamate, benzophenone-3, mexenone, provatene, avobenzone are a few examples of synthetic sunscreen agents, however their usage is limited owing to the adverse side effects such as development of irritant dermatitis, hypersensitivity, allergies and even melanoma [7]. Therefore the usage of natural/herbal sunscreens has gained considerable attention over the recent years and several natural compounds with UV absorption property have been used to substitute for or to reduce the quantity of synthetic sunscreen agents [8].

However it was revealed that the protection is not fully achieved even if the sunscreen is effective in blocking harmful UV rays, thus the high SPF value alone is not adequate for an effective photoprotection. Over the last few years, a significant number of evidence has emerged, indicating that chemically diverse classes of naturally occurring substances are potent in the treatment of several dermatological conditions caused by the chronic exposure to the UV radiation. Polyphenols, flavonoids, terpenes, catechins and alkaloids are compound classes that have been targeted for photochemopreventive activity and curcumine, resveratrol, caffeic acid and quercetin are a few examples of such compounds. It has been suggested that the beneficial effects of these antioxidants might be a successful strategy for diminishing UV-radiation mediated oxidative damage of the skin. For example, the oral administration or topical application of plant extracts such as green and black tea, coffee, *Aloe vera*, cucumber is speculated to be protective against UV-induced erythema, early aging and irradiation-induced cancer [9].

Plants and their products have been systematically used in Sri Lanka for treating illnesses for over thousand years. Among the native flora of Sri Lanka more than 1400 plants are used in indigenous medicine [10] and the literature reveals that large number of plants are extensively used to treat various dermatological diseases as well as to improve complexion [11]. These alternative medications seem promising, although their true effects are not scientifically proven, thus further investigations should be performed to assess the clinical benefits. However, only a handful of scientific evidences are available on bioactivity studies of medicinal plants in Sri Lanka that could lead towards the development of herbal cosmetics. Apart from the study on photoprotective properties of Sri Lankan black tea [12], there has been hardly any report on photoprotective potential of Sri Lankan plants. In order to fulfill this knowledge gap, the present study has focused on evaluation of sunscreening and antioxidant activity of eleven medicinal plants that have been extensively utilized in Sri Lanka for improving complexion and as dermatological therapeutics. Thereby the ethnopharmacological usage of these plant species could also be rationalized.

## Methods

### Plant material

Leaves of *Aporosa lindleyana* (Euphorbiaceae) *Atalantia ceylanica* (Rutaceae)*, Hibiscus furcatus* (Malvaceae)*, Olax zeylanica* (Olacaceae)*, Ophiorrhiza mungos* (Rubiaceae) and whole plants *of Argyreia populifolia* (Convolvulaceae)*, Ipomoea mauritiana* (Convolvulaceae)*, Lasia spinosa* (Araceae)*, Leucas zeylanica* (Lamiaceae) and *Plectranthus zeylanicus* (Lamiaceae) were collected in Gampaha District - Western Province of Sri Lanka in 2013, while seeds of *Mollugo cerviana* (Aizoaceae) was purchased from Ayurvedic retail outlet at the Market Place, Nittambuwa, Sri Lanka. Based on the application in folklore medicine, the above plant parts were specifically selected for the study. The plants were identified by the author (MTN), a botanist, and confirmed based on the books “A Revised Handbook to the Flora of Ceylon: volume – 1-XIII, M.D. Dassanayake & F.R. Fosberg” and “Medicinal plants (indigenous and exotic) used in Ceylon: Volume 1–5 by D.M.A. Jayaweera” and authenticated by comparison with the herbarium specimens at the National herbarium, Royal Botanical Garden, Peradeniya, Sri Lanka. A voucher specimen of each plant is deposited at the Department of Biochemistry, Faculty of Medicine, University of Ruhuna, Sri Lanka.

### Preparation of crude extracts

The plant materials were thoroughly washed and dried in shade (30 °C) for six days. Dried plants were powdered using a domestic grinder (Singer model KA-MIXEE). The powdered materials (10–15 g) were extracted in 300 ml of 70% methanol–water by heating for 2 h at 60 °C. The extracts were evaporated into dryness with the use of rotary evaporator (BÜCHI, R-114, Germany) or a vacuum centrifuge (Thermo, Germany).

#### Evaluation of UV filtering potential

For the determination of UV filtering potential the UV absorption of each extract (1 mg/ml) was measured between 260 and 400 nm using UV-visible spectrophotometer (Shimadzu, UV_1800) and the sun protection factor (SPF) was calculated according the Mansur equation [13].

$$ SP{F}_{spectrophootometric}=CF\times {\displaystyle \sum_{290}^{320}EE}\left(\lambda \right)\times I\left(\lambda \right)\times Abs\left(\lambda \right) $$

Where: EE(λ) – erythemal effect spectrum; I(λ) – solar intensity spectrum; Abs(λ) – absorbance of sunscreen product; CF – correction factor (=10)

The plant extracts were exposed to direct sunlight for 21 days and the UV absorbance was measured at 7^th^ 14^th^ and 21^st^ day to determine the photostability of the extracts.

Two commercially available sunscreen products SC1 (benzophenone-4 and TiO_2_ as active ingredients) and SC2 (*Aloe,* Sandlewood, *Ficus* as active ingredients) were used as reference substances. In addition, an extract prepared with *Aloe vera* (leaves) following the same procedure was used as a control to compare with the UV filtering potential of the selected plant extracts.

## Radical scavenging capability

### DPPH assay

The radical scavenging capability of the extracts was assessed by measuring the reduction of the stable free radical 2,2-diphenyl-1-picrylhydrazyl (DPPH) as described by the method of Blois 1958 [14]. The absorbance was recorded at 517 nm after 30 min incubation of test extracts and DPPH solution under gentle shaking in the dark. The percentage antioxidant activity (AA) was calculated using the following formula and the EC_50_ was determined using GraphPad Prism version 6.01.

$$ AA=\frac{\left(\mathrm{Absorbance}\kern.3em \mathrm{of}\kern.3em \mathrm{the}\kern.3em \mathrm{control}-\mathrm{Absorbance}\kern.3em \mathrm{of}\kern.3em \mathrm{the}\kern.3em \mathrm{sample}\right)}{\mathrm{Absorbance}\kern.3em \mathrm{of}\kern.3em \mathrm{the}\kern.3em \mathrm{control}}\times 100 $$

BHA was used as the positive control and all the measurements were carried out in triplicate.

### ABTS assay

ABTS (2,2′-azino-bis-3-ethylbenzthiazoline-6-sulphonic acid) assay was employed as described by Re et al. 1999 [15]. Briefly, ABTS^.+^ solution (2.5 mM) and potassium persulfate (2 mM) solution in PBS buffer were prepared, mixed in 1:1 ratio and the mixture was allowed to react for 12 h at room temperature in the dark. The absorbance of the ABTS radical solution was adjusted at 0.8 at 734 nm. The ABTS radical solution (150 μl) was mixed with plant sample (50 μl) and the absorbance was measured at 734 nm. All the measurements were carried out in triplicate. Trolox was used as the antioxidant standard and the antioxidant potential was expressed as milimolar of Trolox equivalents per milligram.

### Statistical analysis

All the above experments were perormed in triplicate and the values were given as mean ± S.D.

## Results

### UV filtering potential

Among the eleven extracts *Atalantia ceylanica, Hibiscus furcatus, Leucas zeylanica, Mollugo cerviana, Olax zeylanica* and *Ophiorrhiza mungos* have displayed SPF ≥ 25, which were even higher than two commercial photoprotective creams used as the standard reference substances (Table 1). SPF values of *L. zeylanica*, *O. mungos*, *M. cerviana* and *H. furcatus* were found to be even higher than the SPF of well-known medicinal plant, *Aloe vera*. Interestingly, the real SPF value of the two commercial photoprotective creams were found to be much lower than their labelled SPF value. The most potent six extracts were further subjected to detailed investigations to determine their suitability as components of herbal sunscreens.

**Table 1:** Measured SPF values of the plant extracts (listed in descending order) and commercial sunscreens

Extract	Measured SPF Value
*Leucas zeylanica*	39.8 ± 0.35
*Ophiorrhiza mungos*	39.2 ± 0.92
*Mollugo cerviana*	29.5 ± 0.30
*Hibiscus furcatus*	29.4 ± 0.40
*Atalantia ceylanica*	26.8 ± 0.16
*Olax zeylanica*	24.5 ± 0.47
*Aporosa lindleyana*	21.4 ± 0.90
*Argyreia populifolia*	12.5 ± 0.61
*Plectranthus zeylanicus*	11.5 ± 0.96
*Ipomoea mauritiana*	11.3 ± 0.70
*Lasia spinosa*	8.9 ± 0.57
SC1 (Standard reference)	10.7 ± 0.07 (labeled SPF : 30+)
SC2 ((Standard reference)	18.6 ± 0.01 (labeled SPF : 45)
*Aloe vera* (control)	28.86 ± 0.11

Furthermore *O. mungos* did not display a reduction in the SPF after exposition to direct solar radiation for 21 days. Similarly, *Aloe vera* extract also displayed photostability with SPF values of 24.61 ± 0.82, 23.65 ± 0.08 and 22.84 ± 0.16 on 7^th^, 14^th^ and 21^st^ day respectively.

**Fig. 1:**
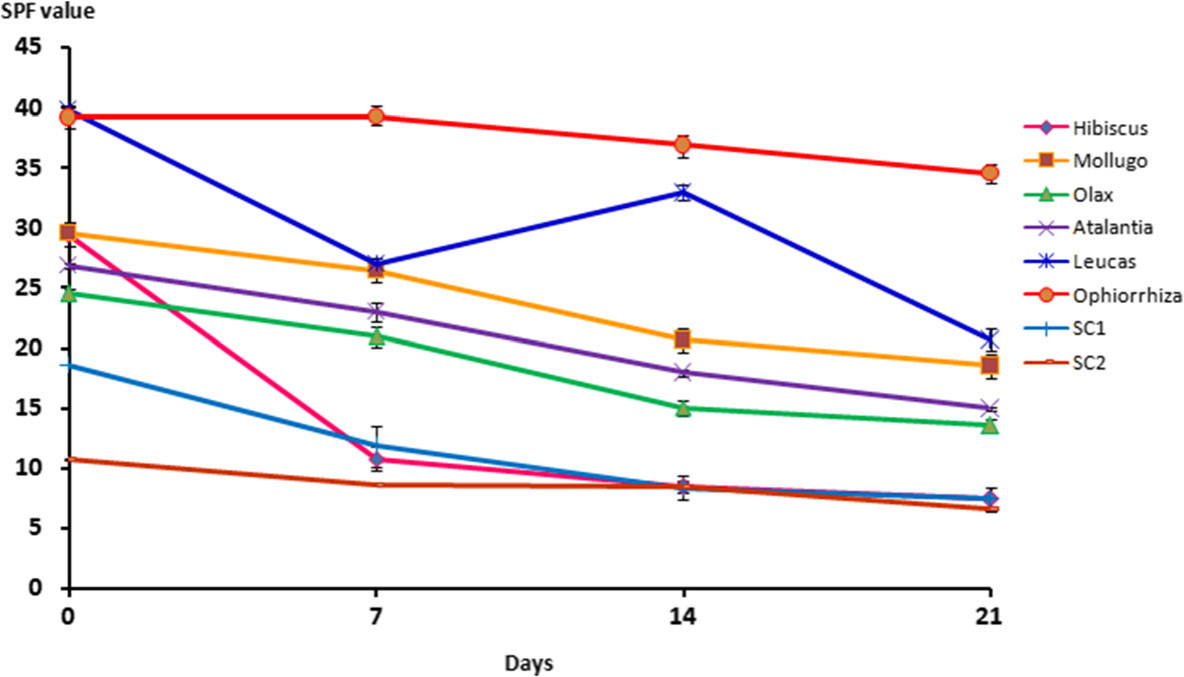
Variation of SPF overtime

**Fig. 2:**
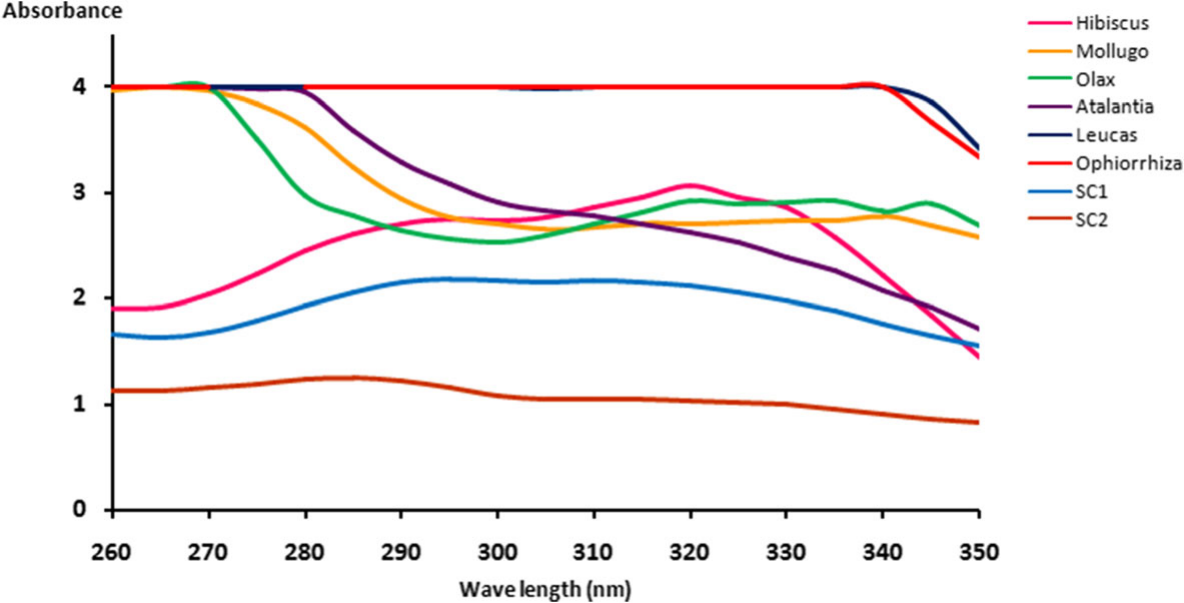
UV absorbance of the extracts

Moreover *L. zeylanica* and *O. mungos* have exhibited high UV absorbance throughout the range of 260–350 nm (Fig. 1 and Fig. 2) - UV absorbance of the extracts). In addition, the maximum absorbance of *H. furcatus* lies within the UV- B range while the other extracts have displayed the maximum absorption in the UV-C range.

#### Radical scavenging capability

The radical scavenging potential was extremely high in *H. furcatus L. zeylanica, M. cerviana, O. zeylanica* and *O. Mungos* extracts according to the DPPH assay, while all the extracts have displayed strong antioxidant activities proving their capacity to scavenge the ABTS radical cation (Table 2).

**Table 2:** Antioxidant activity of the most potent plant extracts

Extract	EC_50_ for DPPH assay	Antioxidant activity as expressed as mM trolox eq/mg
*Atalantia ceylanica*	527 ± 0.83	108 ± 0.003
*Hibiscus furcatus*	32.32 ± 0.79	110 ± 0.01
*Leucas zeylanica*	30.54 ± 0.93	116 ± 0.01
*Mollugo cerviana*	98.72 ± 0.80	100 ± 0.005
*Olax zeylanica*	28.05 ± 0.94	86 ± 0.01
*Ophiorrhiza mungos*	32.49 ± 0.94	109 ± 0.007
BHA (positive control)	23.12 ± 0.96	–

## Discussion

Sri Lanka lies within the equatorial belt, a region which receive ample amount of solar radiation throughout the year. The strength of sunburn-producing UV radiation at a particular place and a time is explained in terms of the UV Index that is defined as the amount of skin damaging UV radiation expected to reach the earth’s surface at the time when the sun is highest in the sky. In Sri Lanka, the UV index varies from 6 to 12 throughout the year [16], that falls within the “high”, “very high” and “extreme” risk categories for which the usage of sunscreens with SPF of 30+ are highly recommended.

SPF value is an indicator that is mentioned in sunscreens which indicates that how much photoprotection provides against UV radiation by sunscreen when it is applied thickness of 2 mg/cm [3] skin [17]. Sunscreen products can be categorized according to their SPF values as minimal (SPF < 12), moderate (SPF 12–30) and high sun protection products (SPF ≥ 30) [18]. According to the SPF value of the sunscreen product, the protection percentage from UV radiation is different. When SPF is 15, it provides > 93% protection against UV- B and SPF +30 provides 97% protection from UV-B. Most of the commercially available sunscreen products are highly effective against UV-B, but not against UV-A [18, 19].

The present investigation reveals that Sri Lankan plant species have a high sunscreening potential that falls within “moderate” and “high” sun protective categories. Thus a sunscreen product developed by these plant extracts would be of high importance specially for the people living in tropical countries. Furthermore, the study reveals that real SPF value was found to be much lower than the labelled value in two commercial sunscreen products and these observations agreed with the previous literature data where the labelled SPF did not correspond to the actual SPF value [20]. Since most chemicals capable of blocking only a narrow region of the UV light, incorporation of several chemicals with each one blocking a different region of UV light would be necessary to offer a broad spectrum UV protection. Therefore the characteristic absorption band exhibited by the extracts of *L. zeylanica* and *O. mungos* in the UV-B and UV-A suggest a possible broad spectrum sunscreening potential, a desirable character of an ideal sunscreen. Usually a sunscreen UV filters may degraded or destroyed over the time or when get exposed to sunlight, thus the development of a sunscreen with photostability is one of the dilemma in the cosmetic industry. In this respect, the photostability displayed by the extract of *O. mungos* indicates that the plant is having a great promise to be developed as a potent topical sunscreen.

Usually the herbal cosmetics contain one or more active sunscreening agents with antioxidant properties in order to achieve a good photoprotective effect. As UV rays absorbed by the skin leads to the production of free radicals such as O_2_^−^, H_2_O_2_, OH^.^, ROO^.^ etc. and deactivate the antioxidant enzymes, thus the incorporation of antioxidants is now widely recommended in sunscreens. Therefore the evaluation of most potent extracts for the antioxidant activity would be important for the development of more effective and efficient sunscreens. As the selected extracts have displayed antioxidant activities, a sunscreen developed with those could boost the body’s defence system against the formation of UV induced free radicals, thus resulting in superior photoprotection. Furthermore, these observations suggest that the extracts might contain secondary metabolites such as polyphenols and flavonoids that are capable of absorbing UV radiation as well as quenching the reactive oxygen species generated in the body. Therefore, the secondary metabolites in the bioactive extracts will be identified by liquid chromatography coupled mass spectrometric (LC-MS) analysis while formulation of a topical sunscreen by incorporation of the most active extracts is currently in progress. Thereafter, more detailed cell based assays are planned for the future to evaluate their suitability for the development of herbal cosmetics and we hope that these findings may further support the ethnopharmacological significance of these plant species as well.

## Conclusions

The preliminary findings of this study reveals that Sri Lankan medicinal plant preparations have a high potential to be used as natural skin care agents due to the high UV absorption properties and the strong antioxidant activities. This would offer an exciting avenue for further research towards the development of herbal cosmetics.

## Abbreviations

ABTS: 2,2′-azino-bis-3-ethylbenzthiazoline-6-sulphonic acid
DPPH: 2,2-diphenyl-1-picrylhydrazyl
LC-MS: Liquid chromatography coupled mass spectrometry
SPF: Sun protective factor
UV: Ultraviolet
